# CAM therapies among primary care patients using opioid therapy for chronic pain

**DOI:** 10.1186/1472-6882-7-15

**Published:** 2007-05-16

**Authors:** Sara Fleming, David P Rabago, Marlon P Mundt, Michael F Fleming

**Affiliations:** 1 ND candidate June 2007, Bastyr University, Seattle, Washington 98122, USA; 2 Department of Family Medicine, University of Wisconsin, Madison, Wisconsin 53715, USA

## Abstract

**Background:**

Complementary and alternative medicine (CAM) is an increasingly common therapy used to treat chronic pain syndromes. However; there is limited information on the utilization and efficacy of CAM therapy in primary care patients receiving long-term opioid therapy.

**Method:**

A survey of CAM therapy was conducted with a systematic sample of 908 primary care patients receiving opioids as a primary treatment method for chronic pain. Subjects completed a questionnaire designed to assess utilization, efficacy and costs of CAM therapies in this population.

**Results:**

Patients were treated for a variety of pain problems including low back pain (38.4%), headaches (9.9%), and knee pain (6.5%); the average duration of pain was 16 years. The median morphine equivalent opioid dose was 41 mg/day, and the mean dose was 92 mg/day. Forty-four percent of the sample reported CAM therapy use in the past 12 months. Therapies utilized included massage therapy (27.3%, n = 248), chiropractic treatment (17.8%, n = 162), acupuncture (7.6%, n = 69), yoga (6.1%, n = 55), herbs and supplements (6.8%, n = 62), and prolotherapy (5.9%, n = 54). CAM utilization was significantly related to age female gender, pain severity income pain diagnosis of neck and upper back pain, and illicit drug use. Medical insurance covered chiropractic treatment (81.8%) and prolotherapy (87.7%), whereas patients primarily paid for other CAM therapies. Over half the sample reported that one or more of the CAM therapies were helpful.

**Conclusion:**

This study suggests CAM therapy is widely used by patients receiving opioids for chronic pain. Whether opioids can be reduced by introducing such therapies remains to be studied.

## Background

Complementary and alternative medicine (CAM) is a treatment method frequently utilized to treat chronic pain syndromes [1-4]. CAM can be defined both by philosophy, as well as by therapeutic modality [5]. CAM practitioners emphasize the holistic, individualistic, empowering, and educational nature of CAM. Many believe the body has the intuitive knowledge to heal itself if given the proper tools, and encourage patients to take responsibility for their own health. In this view, pain is not an entity to be combated, but instead a teacher and guide [4,5].

Examples of CAM therapy used for treating chronic pain include acupuncture, aromatherapy, biofeedback, chiropractic care, energy healing, folk remedy, herbal medicine, homeopathy, hypnosis, imagery, lifestyle diet, massage, megavitamins, naturopathy, osteopathy, relaxation techniques, self-help groups, spiritual healing by others, copper bracelets, and yoga [6,7].

The frequency of CAM therapy use for chronic pain and other medical problems has been reported by a number of researchers. Sherman et al. [8] conducted interviews with 249 patients with chronic low back pain and reported 54% had utilized chiropractic treatment and 38% massage therapy with massage therapy being reported as the most helpful. Population based studies conducted by Eisenberg et al. [6] found that from 1990 to 1997, the use of at least one of 16 CAM therapies in the US grew from 33.8% to 42.1%. Kessler et al. [9] conducted a telephone survey of a stratified sample of 2055 adults and reported increasing demand for CAM therapies across all socioeconomic groups. Characteristics of persons who are most likely to use CAM therapy include adults who are between 35 and 49 years of age, non-African American, college education, incomes above $35,000, poor health status, and having a holistic orientation to health [2].

The efficacy of various CAM therapies for the treatment of chronic pain remains controversial. Acupuncture is probably the most widely tested. Berman et al. [10] conducted an acupuncture trial for the treatment of knee arthritis and found improved function and decreased pain in the acupuncture group compared to sham acupuncture. A meta-analysis conducted by Manheimer et al. [11] analyzed 33 clinical trials designed to test the efficacy of acupuncture for the treatment of low back pain. The report found acupuncture significantly decreased chronic low back pain compared to sham treatment (standard mean difference 0.54, 95%CI, 0.35–0.73). Most of these studies were limited by short follow-up, absence of controlling for potential confounding variables such as pain medication, and small sample sizes.

Prolotherapy is an injection based CAM therapy for chronic musculoskeletal pain in which irritant solutions are injected at tender ligament or tendinous attachments. A recent systematic review review of prolotherapy reported results of 34 case series and 6 randomized controlled trials for a variety of chronic pain conditions; results were often positive but many studies had methodological limitations [12]. While the mechanism of healing remain controversial, studies hypothesize that prolotherapy may promote healing of damaged collagenous tisssue by an anabolic inflammatory response or ablation of pathologic nerve endings [13]. Licciardone et al. [14] reported osteopathic manipulative treatment significantly reduces low back pain at a level greater than expected from placebo effects alone, persisting for at least three months. Cherkin et al. [15] reported massage therapy was associated with significant improvements in chronic low back pain.

The goal of this report is to describe the utilization and self reported efficacy of six CAM therapies (acupuncture, chiropractic manipulation, massage therapy, yoga, prolotherapy and herbs/supplements) by chronic pain patients receiving opioid therapy. In view of the potential adverse effects of chronic opioid therapy (i.e. addiction, mood changes, sedation, accidents, respiratory depression) CAM therapy could provide significant pain relief and minimize the use of opioids.

## Methods

The primary study for which the CAM data was derived was an interview study conducted on a systematic sample of subjects being treated for chronic pain to assess the point prevalence of substance use disorders and opioid addiction. The assessment of CAM therapy utilization was a secondary aim of the study. Additional secondary variables of interest included chronic pain diagnosis, type and dose of opioid, opioid adverse effects, mental health disorders, quality of life, costs, and current physician practice behavior. The data presented in this paper was limited to information relevant to CAM therapy. A number of other papers, based on this data set, are in press with other journals. There is no significant overlap in data presented in these papers.

Subjects were recruited with the help of 235 primary care physicians practicing in eight counties located throughout the state of Wisconsin. These physicians were members of six health care systems including the University of Wisconsin Medical Foundation, Dean Health System, Group Health Cooperative, Medical College of Wisconsin, Aurora Health Care, and Mercy Health Care. Interviews were conducted in primary care clinics and research offices in 2003 and 2004.

Primary Inclusion criteria for the primary group of interest included: a) age between 18 and 81; b) a diagnosis of chronic non-cancer pain; and c) current treatment with chronic opioid therapy by a primary care physician. Chronic pain was defined as continuous pain for at least 3 months. The average duration of pain in the sample was 16 years. Pain severity was not an inclusion criterion. The study was approved by the Human Subjects Committees of the University of Wisconsin, Aurora Medical Foundation, Medical College of Wisconsin, Dean Care Medical Foundation, Meriter Hospital and Mercy Health Care. Financial support for the study was obtained from a National Institute of Drug Abuse (NIDA) R01 grant. There was no industry based financial support used to carry out this study.

### Subject recruitment

Physicians used a number of strategies to identify patients being treated for chronic pain. These strategies included obtaining patient lists from billing records using ICD-9 codes for chronic pain diagnoses, pharmacy records, patient opioid logs maintained by individual physicians and electronic medical record searches. The goal of the recruitment efforts was to enroll 100% of the chronic pain patients receiving opioid prescriptions in each of the 235 physician practices, so as to minimize selection bias. The second step was to mail potential subjects a letter of invitation from their primary care physician. Patients who did not return an "opt-out" post card were contacted by a study researcher by telephone, and if they met the inclusion criteria, were invited to participate in a face-to-face interview.

Written informed consent was obtained at the time of the interview. Of the 1,252 subjects who met the initial study criteria, 1009 participated in a face-to-face interview for a response rate of 80% (1009/1252). Primary reasons for non-participation included lack of time, day care issues, confidentiality concerns and transportation barriers. For this analysis we utilized 908 subjects out the primary sample of 1009 subjects, who reported regular opioid use in the last 3 month and completed all elements of the CAM survey.

### Research instruments

In addition to completing a survey of CAM therapies, five interview schedules and eight additional questionnaires were administered. Interview and questionnaire data used in this report included the Addiction Severity Index (ASI) [16], the Substance Abuse Severity Scale, the chronic pain inventory interview, the 44-question P3 scale, which assesses emotional function [17], and the Treatment Outcomes in Pain Survey (TOPS) which is a modified version of the SF-36 [18]. At the end of the interview each subject was asked to provide a urine specimen that was tested for opioids, methadone, propoxyphene, benzodiazepines, cocaine metabolites, amphetamines, PCP, barbiturates, and cannabinoids.

The CAM survey focused on six CAM therapies including acupuncture, chiropractic therapy, yoga, massage, Prolotherapy, and herbs, and was administered as a questionnaire. The instrument was based on surveys developed by Eisenberg, et al. [2], Astin et al., [1], and Kessler et al. [19]. Participants were also able to write in other therapies used. For each CAM modality patients were asked the following. 1) "Have you used (e.g., acupuncture) for your pain?" (yes or no); 2) "If yes, number of times in the last year?"; 3) "Was the therapy helpful?" (yes or no); 4) "Did your insurance pay for some or all of this therapy?" (yes or no); 5) "Did you have to pay for any of this therapy out of your pocket?" (yes or no); 6) "Total you paid for therapy over the last year?"

### Analysis

Bivariate analysis compared demographic characteristics of participants who reported using or not using CAM therapy in the past 12 months. T-tests and chi-square tests were performed for continuous and categorical measures respectively. Logistic regression analysis was used to model adjusted odds ratios of factors associated with CAM use in the past year and to assess self-reported efficacy of three types of CAM therapy. Continuous variables incorporated into the models included age, education, income, and SF-36 physical and mental component scores. The analysis used increments for each of the continuous variables – age 10-year increments, education four years, income $1000, and SF-36 10 points. The rationale for using increments was to provide constructive odds ratio coefficients related to the strength of the association. Categorical variables in the model included gender, race, diagnosis, substance abuse diagnosis, and cocaine toxicology result. Education, income, and employment, and their corresponding categorical variables were jointly tested for inclusion in the model due to co linearity concerns, with years of education and total income retained. Maximum joint significance was used as criteria for retention.

## Results

Table 1 presents the characteristics of the 404 (44%) participants who reported using a CAM therapy in the past year, compared to 504 participants who reported never using a CAM therapy. The majority of CAM users were women (78.6%) with a mean age of 46.6 years. Compared to non-CAM users, CAM users were more likely to be Caucasian (p < .0.01), have gone to college (54% vs. 38%, p < 0.01), be currently employed (49.9% vs. 35.7%, p < 0.01), and have a higher monthly income ($1,636 vs. $1,322, p < 0.01). CAM users had lower quality of life scores on the SF-36 mental health domain scales (46.5 vs. 49.7, p < 0.05). CAM users were also more likely to have multiple site pain, headache, or upper back and neck pain as their primary pain complaint.

**Table 1:** A comparison of CAM therapy users and non user (The sample consists of 908 subjects taking chronic opioids)

	**CAM user past 12 months**	**No CAM**	**Total**
	**n = 404**	**n = 504**	**n = 908**

Age-Mean	46.6**	50.0**	48.5
Gender, Female	78.6**	61.8**	69.3
Race-White or Caucasian (see footnote 1)	80.9	71.2	75.5
Black or African American	18.4	26.8	23.1
Native American	0.5	1.0	0.8
Hispanic	0.3	1.0	0.7
Education, Mean years (see footnote 2)	13.6**	12.7**	13.1
Education, 12 years or less, %	46.0	62.1	54.9
>12 and <16 years, %	29.2	23.4	26.0
16 or more years, %	24.8	14.5	19.1
Employment-Fulltime or part time	49.6*	35.7*	42.0
Student	1.0	1.0	1.0
Disability	32.3**	45.6**	39.7
Unemployed, looking for work	11.9	10.3	11.0
Marital Status-Married	44.2	41.9	42.9
Widowed	3.5	7.1	5.5
Separated or Divorced	32.0	31.4	31.6
Never Married	20.4	19.6	20.0
Opioids Utilization-Daily	86.6	89.3	88.1
10–29 days per month	13.4	10.7	11.9
Mean dose of Opioid's per day (footnote 3)	94.1 mg	90.4 mg	92 mg
Opioid addiction (DSM-IV 30 day criteria)	3.6%	3.2%	3.4%
Positive Toxicology test for illicit drugs	21.4%	24.1%	22.1%
Pain severity average daily pain (1–10 scale)	4.83	4.77	4.8
Monthly Employment Income, Mean	$845**	$550**	$681
Total Monthly Income, Mean	$1636**	$1322**	$1461
RAND SF-36 Physical Composite Score	33.5	32.7	33.1
RAND SF-36 Mental Composite Score	46.5*	49.7*	48.3
Primary Pain Site,			
Lower back	34.9*	41.3*	38.4
Headache	12.9**	7.5**	9.9
Multiple sites	10.6**	5.9**	8.0
Knee	5.9	6.9	6.5
Neck/upper back	9.2**	5.0**	6.8
Leg	5.0	6.9	6.1
Foot and ankle	3.5*	7.1*	5.5
Shoulder	4.7	4.6	4.6
Hip	4.7	4.4	4.5
Abdominal	3.5	5.2	4.4

*p < .05, **p < .01

1. Using Chi square analysis Caucasians were more likely to use CAM therapy than African Americans. p < 0.01.

2. Using Chi square analysis subjects who utilized CAM therapy were more likely to have attended college than non CAM users. p < 0.01.

3. Opioids utilized by the chronic pain patients in the sampe (i.e. oxycodone, hydrocodone, methadone, fentanyl, meperidine, codeine) were converted to morphine equivalent doses using standard equivalency tables.

Fifty-eight percent of subjects were taking oxycodone with 22.1% receiving long acting oxycodone such as Oxycontin. Other opioids utilized by the sample included hydrocodone (26.2%), morphine (17.2%), codeine (8.6%), duragesic patches (8.3%), methadone (7.5%) and hydrocodone (1.3%). Twenty-three percent of subjects were taking more than one opioid. The mean average daily pain rating on a scale of 1–10 was 4.8. The point prevalence of opioid addiction, using 30 day DSM-IV criteria, was 3.4% (n = 31). This compares to a point prevalence of 1–1.5% in general population samples or about 3 times above the expected frequency. Twenty-two percent (n = 200) had a positive toxicology test for marijuana and/or cocaine.

Table 2 reports the frequency of six types of CAM therapy use, efficacy, and payment data. The most frequent CAM therapy used to treat chronic pain treatment in the last year was massage therapy (27.3%) followed by chiropractic treatment (17.8%) and acupuncture (7.6%). Yoga and prolotherapy were used by about 6% of the subjects. The number of times CAM therapy was utilized in the past year varied by type of therapy, ranging from near daily use (i.e. meditation and herbal medication) to 3.5 sessions of prolotherapy. Insurance coverage provided part of the cost for all types of CAM therapy with the highest reimbursement rates for prolotherapy, chiropractic treatment, acupuncture and massage.

**Table 2:** Frequency of CAM therapy use in lifetime and past year, self-reported efficacy, and cost (The sample consists of 908 subjects receiving chronic opioid therapy)

	Lifetime use	Used in the past year	Number of times used in the past year	Reported CAM therapy was helpful	Insurance coverage paid part of the costs	Paid some cost out of pocket	Mean out of pocket cost in last year
**Response categories:**							
Acupuncture	10.1%	7.6% n = 69	10.5	59.4%	34.1%	68.1%	$522
Chiropractic	23.6%	17.8% n = 162	14.1	80.9%	81.8%	46.3%	$558
Yoga	7.5%	6.1% n = 55	84.0	81.8%	5.9%	55.9%	$162
Massage	34.9%	27.3% n = 248	28.3	90.7%	30.8%	51.3%	$486
Prolotherapy	8.3%	5.9% n = 54	3.5	68.5%	87.7%	18.8%	$365
Herbs/supplements	9.4%	6.8% n = 62	202.8	77.4%	1.3%	89.1%	$212
**Other listed categories:**							
Meditation	1.7%	1.4% n = 13	264.4	84.6%	15.4%	15.4%	$325
Warm water therapy	0.7%	0.7% n = 6	33.3	100.0%	83.3%	50.0%	$147
Craniosacral therapy	0.4%	0.4% n = 4	10.3	75.0%	25.0%	75.0%	$513
Accupressure	0.3%	0.2% n = 2	76.0	100.0%	100.0%	0.0%	$0
All Others (categories listed by 2 or fewer respondents)	1.2%	1.2% n = 11	101.9	81.8%	33.3%	85.7%	$913

**Any CAM**	**64.1%**	**44.5%**	**80.1**	**81.6%**	**45.2%**	**54.7%**	**$353**

Table 2 also presents subjects' self perceived efficacy information. Subjects were asked if they found their CAM therapy helpful. Ninety percent (220 out of 248) of persons who received massage therapy in the last year reported the treatment was helpful. Chiropractic treatment and yoga were similar with 80.9% and 81.3% respectively reporting benefit. This data suggests that when patients did utilize CAM therapy, it was perceived to be helpful most of the time.

Table 3 presents a logistic regression model that tested the association of CAM therapy use in the last 12 months with a number of factors. This table reports the adjusted odds ratio for each variable included in the analysis. The odds ratios in this table are adjusted for all variables listed in table 3, using a logistic regression analysis. This analysis allows us to simultaneously control for the variables listed. For age we elected to use 10 years increments. As noted in the table, the odds of using CAM interventions compared to not using CAM methods were reduced by a factor of 0.73 for every 10 year increment of increasing age. This finding suggests that after adjusting for all the factors in the model, young adults are more likely to use CAM therapy than older adults.

**Table 3:** CAM use in chronic pain patients on opioids by patient characteristics; results from logistic regression analysis *. (The sample consists of 908 subjects receiving chronic opioid therapy)

	Adjusted Odds Ratio **	**95% Confidence Interval**	**p-value**
**Variables:**			
Age (per 10 years) **	**0.73**	**(0.63, 0.85)**	**<.01**
**Female**	**2.27**	**(1.64, 3.14)**	**<.01**
**Education (per 4 yrs)**	**1.41**	**(1.10, 1.82)**	**<.01**
**Race:**			
White	1.00	---	
African American	0.82	(0.57, 1.18)	.588
Other	0.45	(0.12, 1.75)	.308
**Pain Severity**	**1.11**	**(1.01, 1.22)**	**.026**
SF-36 physical component (per 10 points)	1.05	(0.95, 1.15)	.340
SF-36 mental component (per 10 points)	0.93	(0.85, 1.01)	.092
**Total income (per $1000)**	**1.18**	**(1.06, 1.31)**	**.05**
Primary pain site:			
**Neck/upper back**	**2.27**	**(1.24, 4.14)**	**.01**
**Multiple sites**	**1.81**	**(1.05, 3.11)**	**.033**
Headaches	1.42	(0.84, 2.40)	.187
Lower back	1.00	(0.72, 1.39)	.995
Others	1.00	---	
**Cocaine tox screen negative**	**1.84**	**(1.01, 3.36)**	**.047**

* This table reports the adjusted odds ratio for each variable included in the analysis. The odds ratios in this table are adjusted for all variables listed in table 3, using a logistic regression analysis.

**For age we elected to use 10 years increments. As noted in the table the odds of using CAM interventions compared to not using CAM methods were reduced by a factor of 0.73 for every 10 year increment of increasing age. This finding suggests that after adjusting for all the factors in the model, young adults are more likely to use CAM therapy than older adults.

The model found a statistically significant association between CAM therapy and gender (OR 2.27: 1.64, 3.14), age (OR 0.73: 0.63,0.85), education (OR 1.41: 1.10,1.82), pain severity (OR 1.11: 1.01,1.22), income (OR 1.18: 1.06,1.31), neck and upper back pain location (OR 2.27: 1.24,4.14), multiple pain sites (OR 1.81: 1.05, 3.11), and negative cocaine use (OR 1.84: 1.01, 3.36).

## Discussion and conclusions

This report provides new information on CAM therapy in a primary care sample of patients being treated with chronic opioid therapy. The sample is unique from a number of perspectives. First, patients in this study report the average duration of chronic pain was 7.1 years. Second, using a 1–10 analogue, the average daily pain severity was 4.8. Third, subjects are severely disabled, with 40% receiving social security disability and less than 42% able to work full- or part-time. Fourth, the majority of patients used opioids on a daily basis at an average dose of 92 mg. The doses ranged from 2.5 mg to 640 mg per day. Fifth, the sample includes a wide range of chronic pain diagnosis. Sixth, the sample was recruited from a diverse number of primary care practices located throughout Wisconsin and included rural settings, a large urban area, and small cities.

The study found that 44.2% of the sample used a CAM therapy in the year prior to the study with massage therapy and chiropractic treatment used most frequently. This is generally consistent with use in the general US population; Eisenberg et al. [5] reported 42.1% of the US population had used a CAM therapy in 1997. Direct comparison to national studies is difficult as we limited our survey to six primary CAM therapies. The national studies conducted by Eisenberg included 16 different CAM therapies. We would expect higher rates in our sample if we had included all potential therapies, suggesting patients in our sample may have higher utilization rates if we had used a more comprehensive list. Our findings are also similar to the prevalence of CAM use in a population of chronic pain patients with spinal cord injuries who reported 40.3% used these alternative treatments in the past year [20].

Our study was consistent with other national trends. Users of CAM therapies in this study were more likely to be slightly younger, better educated and have slightly higher income than non-users [7]. New findings in this report suggest CAM therapy was more frequently used by patients with more severe pain, those with neck and upper back pain, and persons with negative toxicology for cocaine use.

This study raises a number of clinical and research questions that we are unable to address with the retrospective design used for our study. What is the role of CAM therapy in patients receiving opioids? What type of CAM therapies should be utilized in patients using opioids? For which type of chronic pain syndrome are CAM therapies likely to be useful in patients using opioids? What are reasonable outcomes to expect from CAM therapy and opioids? Should patients be started on opioids before all appropriate CAM therapies have been exhausted? With the increasing use of opioids and CAM therapies for chronic pain clinicians need evidence to try to answer these questions. Naturalistic prospective cohort studies and randomized control trials are needed to help physicians, health policy makers and patients.

The study has a number of strengths including a large sample size (n-908), high participation rates (78%), a diverse population or rural and urban adults (25% minority), a large number of pain diagnosis, patients on chronic opioid therapy, state of the art research procedures, extensive information on potential confounders such as depression and substance abuse, and findings of interest to clinicians. Limitations include the retrospective nature of the data, limited information on CAM therapy reported by each patient, and dependence on patient self report questionnaires and interviews. In addition, due the small number of subjects who used individual CAM therapies, we were not able to assess which factors predict a subject's positive response to these therapies.

While Table 2 provides preliminary data that suggests many patients with severe chronic pain find CAM therapy helpful, our ability to determine the efficacy of CAM therapies was limited to self-report. We did not have the resources to conduct an extensive interview with research subjects. In addition, we used a single question to assess patient self-reported efficacy, (i.e. was CAM therapy helpful (yes or no)?), which did not take multiple variables into account, such as indications for treatment, duration of treatment, provider variability, or dosage and quality of herbs/supplements. We are addressing a wide range of CAM therapies, each with great variability. Additionally, many of the CAM therapies can potentially have added effects on reducing pain and assisting patients to live more normal lives.

### Recommendations

Chronic opioid therapy has become a common treatment method for severe chronic pain. Given the well-known side-effects and risk profiles of chronic opiod therapy, including addiction, aberrant drug behaviors, cognitive effects and respiratory depression, clinicians are typically interested in the lowest effective dosing strategy. Our data suggest that patients use and may benefit from specific CAM therapies. Clinicians treating chronic pain patients may consider use of these therapies as initial or adjunctive treatment. Whether opioid use in chronic pain patients can be reduced by introducing CAM therapies remains to be studied, but is of interest especially if physicians are considering utilizing opioid treatment for the remainder of the patient's life.

## Competing interests

The author(s) declare that they have no competing interests.

## Authors' contributions

All of the authors have read and approved the final manuscript. SF is the primary author and wrote the manuscript, assisted with the analysis, and decided on the major conclusions of the study. DR was a second author and helped design the instrument, wrote some of the manuscript and made substantial comments in his role as a physician and CAM researcher. MM was the primary statistician on the study and conducted all of the analysis and helped write the manuscript. MFF was the PI on the NIH funded study and participated in all aspects of the paper including the research design, data collection, analysis and writing of the manuscript.

## Pre-publication history

The pre-publication history for this paper can be accessed here:


